# Endoscopic transorbital approach for repair of spontaneous CSF leak and frontal sinus encephalocele

**DOI:** 10.3171/2025.1.FOCVID24153

**Published:** 2025-04-01

**Authors:** Iñigo L. Sistiaga, Gregorio Catalán-Uribarrena, Francisco Valcárcel, Yuly Garcia-Orozco, Javier Altamirano-Cruz, Mikel Oñate, Iñigo Pomposo

**Affiliations:** Departments of 1 Neurosurgery, University Hospital Cruces, Bilbao, Basque Country, Spain and; 2 Otorhinolaryngology–Head and Neck Surgery, University Hospital Cruces, Bilbao, Basque Country, Spain;; 3 Department of Neurosurgery, Northwell Health, Donald and Barbara Zucker School of Medicine at Hofstra/Northwell, Hempstead, New York; and; 4 University of The Basque Country, Leioa, Basque Country, Spain

**Keywords:** endoscopic, transorbital, CSF leak, encephalocele, minimally invasive, skull base

## Abstract

CSF leaks due to anterior skull base defects, particularly those involving the posterior wall of the frontal sinus, are complex and often require surgical intervention. The endoscopic transorbital approach provides a direct, minimally invasive method with enhanced access and improved working angles. The authors present a video of a 41-year-old female with a spontaneous CSF leak and frontal sinus encephalocele who underwent endoscopic transorbital repair. This approach effectively controlled the defect, resulting in the resolution of the CSF leak with no recurrence at 1-year follow-up. The endoscopic transorbital approach is a reliable surgical option for these challenging cases.

The video can be found here: https://stream.cadmore.media/r10.3171/2025.1.FOCVID24153

## Transcript

In this video, we present a case of an endoscopic transorbital approach, performed through a superior eyelid incision, to repair a spontaneous, idiopathic anterior skull base defect resulting in a cerebrospinal fluid (CSF) leak and frontal sinus encephalocele.

### 0:38 Clinical History.

The patient is a 41-year-old female with a medical history significant for obesity and hypertension, presenting to our institution with acute CSF rhinorrhea. She reported no history of trauma or prior surgeries. Her neurological examination was unremarkable, with no additional symptoms reported and no papilledema was noted.

### 1:07 Preoperative Imaging.

Preoperative imaging included a CT scan and MRI, which revealed a complex defect in the anterior skull base. Specifically, there was a defect in the posterior wall of the frontal sinus, with an encephalocele extending into it. This coronal view clearly demonstrates the bony defect and the encephalocele’s protrusion into the lateral portion of the sinus, located just above the superior wall of the orbit. Notably, the frontal sinus appeared generously pneumatized.

Given the absence of trauma or other precipitating events, this CSF fistula was classified as spontaneous, likely secondary to intracranial hypertension. Conservative management with rest and acetazolamide was attempted but proved unsuccessful, leading to the decision for surgical intervention 3 weeks after the initial presentation.

### 1:52 Rationale for Approach Selection.

Multiple surgical approaches were considered for this case. Given the location of the defect at the anterior cranial base, extending into the frontal sinus, an endoscopic approach was preferred over a transcranial route. While the endonasal approach was also considered, the lateral placement of the defect made access challenging due to the excessive angle required. The endoscopic transorbital approach, offering an ideal lateral-to-medial and anterior-to-posterior trajectory, provided reduced working distances and improved instrumentation angles. This facilitated a direct, minimally disruptive pathway to the frontal sinus after drilling the thin superolateral orbital roof.^1–4^ Therefore, the endoscopic transorbital approach was selected.

### 2:33 Patient Positioning.

The patient was positioned supine with the head supported in a neutral position on a horseshoe headrest. The entire procedure was a collaborative effort by our skull base team, composed of neurosurgery and ENT specialists who routinely perform endoscopic endonasal approaches.

### 3:01 Skin Incision.

The incision was made along the lateral aspect of the superior eyelid, approximately 8 mm above the horizontal palpebral fissure, using a 10 blade scalpel. It was then deepened through the orbicularis oculi muscle with a monopolar microdissection needle, carefully traversing the plane between the pretarsal and preseptal portions.^5^ Blunt dissection of the preseptal plane was performed to expose the lateral orbital rim, followed by an incision of the periosteal layer.^6^

### 3:22 Access and Drilling.

The subperiosteal layer was identified and followed to expose the superolateral orbital wall under endoscopic visualization. A malleable retractor was used to gently displace the orbital contents inferomedially. Neuronavigation guided us to the area of the superior orbital wall closest to the defect. The superior orbital wall was then drilled using a 3-mm diamond burr.

### 4:13 Exposure.

Upon entering the frontal sinus, we enlarged the entry just enough to provide posterior access. We then carefully explored the area and gently removed the mucosa surrounding the defect. Here we can see the frontal sinus mucosa removal. We proceeded by removing the mucosa until the encephalocele was identified. At this point, we observe the encephalocele occupying the frontal sinus and implanting into its posterior wall, specifically in the most posterior and lateral regions. Adjacent supraorbital ethmoid cells were also occupied by herniated brain tissue.

Since the encephalocele was not reducible, it was coagulated and resected, allowing us to visualize the underlying bony defect. Here, we can clearly see the defect in the anterior cranial fossa, with an associated dural tear and exposure of the arachnoid.

### 7:06 Reconstruction.

In this case, due to the size of the defect, a two-layer reconstruction was performed using an inlay fat graft and an overlay collagen graft. Alternative techniques, such as securing the fat graft with fibrin glue or supporting it with fascia lata, were considered but deemed unnecessary. Fat was harvested from the lateral aspect of the left thigh and placed intradurally. Subsequently, the area was sealed with a collagen pad graft for additional coverage. The graft was gently secured in place, using a cottonoid to ensure proper positioning. Here, we can see the final result.

### 8:41 Closure.

Finally, closure of the approach was performed in a multilayer fashion. The periosteum and orbicularis muscle were sutured using 4-0 Vicryl. The skin was then closed with 4-0 Prolene.

### 9:16 Outcome.

The postoperative CT scan demonstrated resolution of the encephalocele occupying the frontal sinus. The patient remained in the ICU overnight and was subsequently transferred to the floor. She was kept on bed rest for a total of 2 days as a precaution. The CSF leak resolved following the intervention, and at 1 year of follow-up, no recurrence of the fistula has been observed.

To address the suspected intracranial hypertension, an intracranial pressure monitoring study was performed several months after the surgery, which confirmed the diagnosis. Subsequently, a ventriculoperitoneal shunt was placed.

## Disclosures

The authors report no conflict of interest concerning the materials or methods used in this study or the findings specified in this publication.

## Author Contributions

Primary surgeon: Catalán-Uribarrena, Valcárcel. Assistant surgeon: Sistiaga, Altamirano-Cruz, Oñate. Editing and drafting the video and abstract: Sistiaga, Catalán-Uribarrena, Garcia-Orozco. Critically revising the work: Sistiaga, Catalán-Uribarrena, Garcia-Orozco, Oñate. Reviewed submitted version of the work: Sistiaga, Valcárcel, Oñate, Pomposo. Approved the final version of the work on behalf of all authors: Sistiaga. Supervision: Catalán-Uribarrena, Valcárcel, Pomposo. Patient care: Sistiaga, Altamirano-Cruz, Catalán-Uribarrena.

## Supplemental Information

### Patient Informed Consent

The necessary patient informed consent was obtained in this study.
